# A Manual of Auscultation and Percussion

**Published:** 1845-12

**Authors:** 


*A Manual of Auscultation and Percussion.* By M. Barth, Agrégé to the Faculty of Medicine of Paris, &c. &c., and M. Henry Roger, Physician to the Bureau Central of the Parisian Hospitals, &c. &c. *Translated, with additions,* by Francis G. Smith, M. D., Lecturer on Physiology in the Philadelphia Medical Association, &c. &c. 12mo. pp. 160. Philadelphia, 1845.

The "Practical Treatise on Auscultation and Percussion" of Messrs. Barth and Roger is regarded in France as a standard vade-mecum for the medical student and practitioner. It is fuller, however, than is necessary; and therefore Dr. Smith has judiciously translated the *Resumé* to the second edition, which contains enough to be an excellent accompaniment and adjuvant to the practitioner; and is especially valuable to him, in consequence of its being divested of all unnecessary detail. We can recommend it as a good pocket instructor—*Taschenbuch*—which will never mislead. The translator is well fitted for this or any similar vocation. He is one of our best informed and most estimable physicians; highly valued by all who know him; and engaged in a career of usefulness, both as a practitioner, and an instructor in the "Philadelphia Medical Association," the lecturers in which are energetically and ably employed in teaching the different departments of medicine during the summer.

Dr. Smith has added many useful notes, which are well worthy the attention of observers.

